# Direct and Indirect Effects of Rotavirus Vaccination: Comparing Predictions from Transmission Dynamic Models

**DOI:** 10.1371/journal.pone.0042320

**Published:** 2012-08-13

**Authors:** Virginia E. Pitzer, Katherine E. Atkins, Birgitte Freiesleben de Blasio, Thierry Van Effelterre, Christina J. Atchison, John P. Harris, Eunha Shim, Alison P. Galvani, W. John Edmunds, Cécile Viboud, Manish M. Patel, Bryan T. Grenfell, Umesh D. Parashar, Ben A. Lopman

**Affiliations:** 1 Department of Ecology and Evolutionary Biology, Princeton University, Princeton, New Jersey, United States of America; 2 Fogarty International Center, National Institutes of Health, Bethesda, Maryland, United States of America; 3 Department of Epidemiology of Microbial Diseases, Yale School of Public Health, New Haven, Connecticut, United States of America; 4 Department of Biostatistics, Institute of Basic Medical Sciences, University of Oslo, Oslo, Norway; 5 Department of Infectious Diseases Epidemiology, Norwegian Institute of Public Health, Oslo, Norway; 6 Global Vaccine Development, GlaxoSmithKline Biologicals, Wavre, Belgium; 7 Infectious Diseases Epidemiology Unit, Department of Epidemiology and Population Health, London School of Hygiene and Tropical Medicine, London, United Kingdom; 8 Centre for Infections, Department of Gastrointestinal, Emerging and Zoonotic Infections, Health Protection Agency, London, United Kingdom; 9 Deparment of Epidemiology, Graduate School of Public Health, University of Pittsburgh, Pittsburgh, Pennsylvania, United States of America; 10 Epidemiology Branch, Division of Viral Diseases, National Center for Immunization and Respiratory Diseases, Centers for Disease Control and Prevention, Atlanta, Georgia, United States of America; Massey University, New Zealand

## Abstract

Early observations from countries that have introduced rotavirus vaccination suggest that there may be indirect protection for unvaccinated individuals, but it is unclear whether these benefits will extend to the long term. Transmission dynamic models have attempted to quantify the indirect protection that might be expected from rotavirus vaccination in developed countries, but results have varied. To better understand the magnitude and sources of variability in model projections, we undertook a comparative analysis of transmission dynamic models for rotavirus. We fit five models to reported rotavirus gastroenteritis (RVGE) data from England and Wales, and evaluated outcomes for short- and long-term vaccination effects. All of our models reproduced the important features of rotavirus epidemics in England and Wales. Models predicted that during the initial year after vaccine introduction, incidence of severe RVGE would be reduced 1.8–2.9 times more than expected from the direct effects of the vaccine alone (28–50% at 90% coverage), but over a 5-year period following vaccine introduction severe RVGE would be reduced only by 1.1–1.7 times more than expected from the direct effects (54–90% at 90% coverage). Projections for the long-term reduction of severe RVGE ranged from a 55% reduction at full coverage to elimination with at least 80% coverage. Our models predicted short-term reductions in the incidence of RVGE that exceeded estimates of the direct effects, consistent with observations from the United States and other countries. Some of the models predicted that the short-term indirect benefits may be offset by a partial shifting of the burden of RVGE to older unvaccinated individuals. Nonetheless, even when such a shift occurs, the overall reduction in severe RVGE is considerable. Discrepancies among model predictions reflect uncertainties about age variation in the risk and reporting of RVGE, and the duration of natural and vaccine-induced immunity, highlighting important questions for future research.

## Introduction

Rotavirus is the leading cause of severe diarrhea in children, representing a major source of morbidity and mortality worldwide. The recent development and licensing of two vaccines, Rotarix (GlaxoSmithKline Biologicals; Rixensart, Belgium) and RotaTeq (Merck & Co; Whitehouse Station, NJ), provide a novel means of controlling rotavirus. Early evidence from developed countries that have introduced rotavirus vaccination into their national immunization program strongly supports the direct and indirect benefits of vaccination [1], [2], [3], [4], [5], [6]. However, the enormous potential these vaccines hold for preventing morbidity and mortality from rotavirus-associated gastroenteritis (RVGE) has yet to be fully realized as many countries, particularly those in high mortality settings of Asia and Africa, have yet to implement routine rotavirus immunization programs.

In response to the advent of rotavirus vaccines, there has been a recent surge in the development of mathematical models for the transmission dynamics of rotavirus [7], [8], [9], [10], [11], [12], [13]. Such models are essential for understanding the full epidemiological impact of vaccination, including the potential indirect effects, i.e. “herd protection" for vaccinated and unvaccinated individuals resulting from reduced transmission of rotavirus in the community. The models can assist in early decision-making and provide insight into the potential cost-effectiveness of vaccination by projecting vaccine-induced changes in the epidemiology of RVGE over time.

Models for the transmission dynamics of rotavirus are structured based on studies of the natural history of infection and immunity [14], [15], [16], [17], [18], [19], [20], [21]. However, such data can have multiple interpretations, and information linking individual-level data on the course of infection to the between-person transmission of rotavirus is lacking. These issues have led to variation in the structure of mathematical models for rotavirus and differences in the parameter estimates that are used to implement the models. Although studies typically explore the sensitivity of model outcomes to the input parameters, the sensitivity of outcomes to variation in *model structure* is rarely addressed for the dynamics of any infection [22], [23], [24], [25], [26], and has not previously been explored for rotavirus.

Whereas some models predict that the reduction in RVGE due to vaccination will exceed estimates derived only from the direct effects of vaccination [8], [11], [12], [13], other models predict that there will be little or no long-term indirect effects of vaccination [7], [9], [10]. All of these outcomes have been evaluated on different time scales using models calibrated to data from different countries. Given these varied predictions, it is essential for policymakers to understand the sources of uncertainty in the formulation and parameterization of rotavirus models in order to have a sound basis for evaluating the expected benefits of introducing rotavirus vaccines into national immunization schedules. Public health officials also need to understand the sources of uncertainty as they assess the impact of new rotavirus vaccine programs and use models to aid in the interpretation of post-introduction disease dynamics, particularly when seasonality or age-patterns of disease appear to change.

We brought together five groups that have previously developed dynamic mathematical models for the transmission of rotavirus [7], [8], [9], [10], [11], [12], [13]. We compared five model structures and their statistical validation against epidemiological data. We adapted the models by making common parameter assumptions where possible (Table S1) and fitting to age-stratified reports of laboratory-confirmed RVGE in England and Wales (E&W), taking advantage of nationwide surveillance data collected for an extended period of time which also included detailed information on age of cases, and had been calibrated against community incidence (i.e. there is an estimate of the reporting fraction, which incorporates the likelihood that an ill person will present to a general practitioner, be tested for rotavirus, and that a positive test will be reported to the surveillance system) [27], [28], [29]. Furthermore, our evaluation of the magnitude of the indirect effects of vaccination may be useful in informing cost-effectiveness analyses and future evaluations of whether to introduce rotavirus vaccines into the routine immunization schedule in E&W. For each best-fit model, we explored the impact of vaccination on both the short-term dynamics and long-term levels of rotavirus incidence under a variety of assumptions about vaccine-induced protection and coverage. This approach provided a unique opportunity to examine the range of model projections regarding the impact of rotavirus vaccination under a variety of model structures.

## Methods

### The Models

The five models for the transmission dynamics of rotavirus we explored follow an SIS- (susceptible-infectious-susceptible) or SIRS-like (susceptible-infectious-recovered-susceptible) compartmental framework (Table 1). All the models assume that infants are protected by maternal immunity at birth, which wanes after a mean period of approximately 3 months (Table 2). Compartmental diagrams of the models can be found in the Supporting Information (Figure S1). Fixed model parameters are described in Table 2, while parameters estimated from fitting the models to the E&W data are described in Table 3 (see Text S1).

**Table 1:** Description of key features of the five models.

Model A	Model B	Model C	Model D	Model E
Risk of infection and severity of RVGE depend on age	Risk of infection and severity depend on the number of previous infections	Risk of infection and severity depends on age and the number of previous infections; short delay between infection and onset of infectiousness	Risk of infection and severity depends on the number of previous infections	Following infection, individuals develop full immunity or become susceptible again
Temporary immunity following infection	Temporary immunity following infection	Temporary immunity following infection	No period of full immunity following infection	Probability of developing full immunity depends on the number of previous infections
Severe and mild RVGE are tracked separately and vary in infectiousness; asymptomatic infections do not transmit	After 2 infections, subsequent infections are less infectious and not reported	After 2 infections, subsequent infections are less infectious and not reported	After 4 infections, all individuals develop full immunity (that may wane)	After 4 infections, all individuals develop full immunity (that may wane); asymptomatic infections do not transmit
Only severe RVGE cases are reported	Only severe RVGE cases are reported	Only severe RVGE cases are reported	Mild and severe RVGE cases are reported; reporting rate depends on age (<5 or ≥5 years old)	Mild and severe RVGE cases are reported

**Table 2:** Fixed parameter values for the five models.

Parameter	Model A	Model B	Model C	Model D	Model E
Duration of maternal immunity	13 weeks	13 weeks	13 weeks	13 weeks	13 weeks
Duration of incubation period	NA	NA	1 day	NA	NA
Duration of infectiousness					
*First infection*	7 days (severe)	7 days	7 days	7 days	7 days
*Subsequent infections*	3.5 days (mild)	3.5 days	3.5 days	3.5 days	7 days
Relative risk of infection following:					
*First infection*	NA	0.62	0.62	0.62	0.62
*Second infection*		0.37	0.37	0.37	0.37
*Third infection*		0.37	0.37	0.37	0.37
Proportion of infections with any RVGE (severe RVGE)					
*First infection*	0.76 (0.24) for	0.47 (0.13)	0.47 (0.13)	0.47 (0.13)	0.47 (0.13)
*Second infection*	<5 yr olds	0.25 (0.03)	0.25 (0.03)	0.25 (0.03)	0.25 (0.03)
*Third infection*	Estimated for ≥5	0.20 (0)	0.20 (0)	0.32 (0)	0.32 (0)
*Fourth infection*	yr olds	NA	NA	0.20 (0)	0.20 (0)
Relative infectiousness (compared to first infection)					
*Second infection*	0.5 for mild vs severe RVGE;	0.5	0.5	0.5	Only individuals with any RVGE
*Subsequent infections*	asymptomatic infections do not transmit	0.2	0.2	0.2	transmit (see above)
Duration of complete immunity	1 year	1 year	1 year	NA	NA
Type of cases reported	Severe RVGE	Severe RVGE	Severe RVGE	Any RVGE	Any RVGE

**Table 3:** Parameters estimated by fitting models to data on laboratory-confirmed RVGE cases in England and Wales.

Parameter	Symbol	Model A	Model B	Model C	Model D	Model E
Duration of immunity to symptomatic infection	1/*ω*	NA*	280 years	A = 7.31e-9, B = 0.228†	833 years	201 years
Amplitude of seasonality in transmission	*b*	0.064	0.057	0.040	0.046	0.052
Seasonal offset	*φ*	0.089	0.377	0.209	0.014	0.237
Age-specific risk of infection	*q_i_,* for age group *i*, as specified	0.083 (<1 y), 0.065 (1 y), 0.017 (2 y), 0.006 (3 y), 0.003 (4–65 y), 0.025 (≥65 y)	0.291 (all age groups)	0.402 (all age groups)	0.562 (<1 y), 0.718 (1 y), 0.344 (2 y), 0.144 (3 y), 0.077 (4 y), 0.068 (≥5 y)	0.890 (all age groups)
Proportion of cases with severe RVGE in ≥5 yr olds	*d_i_*	0.015	NA	NA	NA	NA
Reporting fraction	*r*	0.064	0.122	0.123	0.024 (<5 y), 0.005 (≥5 y)	Fixed at 0.029
Basic reproductive number	*R*_0_	1.23	18.2	17.6	5.03	26.2
Number of parameters estimated	*k*	10	5	6	12	4
**Akaike information criterion (AIC)**		71,990	84,977	76,303	83,912	66,697

* NA = Not applicable.

† An exponential distribution was used to describe increasing probability for reported symptomatic infection with age. With probability p(a) = A*exp(a*B) exposed individuals in “later infection" are moved to exposed group of second infection. The remaining 1-p(a) continues to the “later" infection group. The age a was chosen as the midpoint of the various age groups.

Model A is based on Shim et al (2009) [12] and Atkins et al (2012) [8] and follows an SIRS framework with two levels of disease severity, one representing severe RVGE and the other mild RVGE. These two levels of severity were assumed to have different infectious periods and levels of infectiousness (Table 2). Asymptomatic cases of rotavirus infection were assumed not to play a role in transmission. Reported cases were assumed to represent a proportion of the severe RVGE cases, whereas mild cases were assumed to go unreported (Table 2). Following infection, it was assumed that there is a period of temporary immunity, after which individuals re-enter the fully susceptible class. However, the risk of infection was assumed to vary with age, and was estimated by fitting the model to the E&W data (Table 3). After 5 years of age, it was assumed that the proportion of cases with severe compared to mild RVGE declines; this parameter was estimated by fitting to the data (Table 3).

Model B is based on Pitzer et al (2009) [10] and follows an SIRS-like structure with different levels of susceptibility used to represent the declining risk of infection following one, two or more infections. The protection conferred by primary and secondary infections against subsequent infection was parameterized based on data from the Velazquez et al (1996) Mexico cohort study [17] (Table 2). Primary, secondary, and subsequent infections were assumed to have different durations and different levels of infectiousness (Table 2). Only a proportion of primary and secondary infections were assumed to develop severe RVGE, a fraction of which are subsequently reported. The proportion of cases developing severe RVGE was parameterized from the Mexico cohort study data (Table 2); the reporting fraction was estimated by fitting the model to the E&W data (Table 3). Following infection, it was assumed that there is a period of temporary complete immunity in which individuals cannot be reinfected (Table 2). Afterwards, individuals become susceptible again, but have partial immunity such that subsequent infections occur at a reduced rate, as mentioned above. To account for a possible increase in the risk of symptomatic infection in older age groups, it was assumed that partial immunity may wane over time; the rate of waning of partial immunity was estimated by fitting to the E&W data (Table 3).

Model C is based on de Blasio et al (2010) [9] and follows a structure similar to that of Model B. However, Model C also allowed for a 1-day incubation or “exposed" period following infection prior to the onset of infectiousness (Table 2). Thus, it follows an SEIRS-like structure. In addition, the waning of partial immunity to symptomatic infection among re-infected individuals with more than two past infections was assumed to be age-dependent rather than occurring at a constant rate (Table 3).

Model D is based on Van Effelterre et al (2009) [13] and follows an SIS-like structure. It was assumed that there is a decreased risk of reinfection following one, two or three infections (similar to Models B and C) and full immunity following four infections, but there was no temporary complete immunity following infection (Table 2). Furthermore, the risk of infection was assumed to be age-dependent (similar to Model A) and the full immunity gained after four infections was allowed to wane over time. The age-specific risk of infection and the rate of waning of immunity were estimated by fitting the model to the E&W data (Table 3). Primary, secondary, and subsequent infections were assumed to have different durations and different levels of infectiousness (Table 2). Reporting was assumed to reflect any (severe and mild) RVGE rather than just severe cases. Again, a proportion of first, second, third, and fourth infections were assumed to develop mild and/or severe RVGE, in accordance with data derived from Velazquez et al (1996) [17] (Table 2). The fraction of any RVGE cases reported was assumed to differ between individuals <5 years of age and those ≥5 years of age, which were estimated by fitting the model to the E&W data (Table 3).

Model E is based on Atchison et al. (2010) [7] and follows a hybrid SIS-SIR-like structure. It was assumed that some individuals develop long-term immunity following infection, whereas others become susceptible to reinfection at the same rate as fully susceptible individuals. Thus, natural immunity was assumed to be “all-or-nothing" as opposed to “leaky" [30], [31]. The fraction of individuals becoming immune or susceptible to reinfection, as well as the proportion of infections developing mild and/or severe RVGE, was parameterized based on data from Velazquez et al (1996) [17] (Table 2). It was assumed that the duration of infectiousness is the same for all infections, but that only symptomatic RVGE cases are infectious (Table 2). Reporting was assumed to reflect any RVGE cases (like Model D), with the reporting fraction based on a study of national surveillance and reporting practices in E&W [28]. Immunity to reinfection could wane over time among older individuals, with the rate of waning estimated by fitting the model to the E&W data (Table 3).

### Model Fitting

We estimated several (non-fixed) model parameters and statistically validated and compared our models by fitting each model to age-stratified reports of laboratory-confirmed rotavirus infection from England and Wales [27], [28], [29]. Data on the number of rotavirus-positive specimens reported by diagnostic laboratories to the Health Protection Agency from January 1999 to June 2009 were stratified by calendar week and age in the following 19 age groups: 0–1 month, 2–3 months, 4–5 months, 6–7 months, 8–9 months, 10–11 months, 12–13 months, 14–15 months, 16–17 months, 18–19 months, 20–21 months, 22–23 months, 2 years, 3 years, 4 years, 5–24 years, 25–44 years, 45–64 years, and ≥65 years old. We assumed that transmission-relevant mixing reflects the self-reported number of physical contacts among members of different age groups in E&W (see Text S1) [32]; the probability of transmission given exposure to an infectious contact was estimated for each model by fitting to the E&W rotavirus data. Seasonality in the transmission rate was modeled using a sinusoidal function, the parameters of which were estimated by fitting to the E&W data (see Text S1). To ensure that baseline demographic conditions were equivalent across models, we assumed a constant population size and birth rate through time (both before and after the introduction of the vaccine) equal to estimates for E&W in 2008 (population size of 54.5 million and birth rate of 13.0 per 1,000 population = 708,700 births per year). Deaths occurred only upon exiting the last age group (≥65 years old), leading to an approximately square age distribution that is roughly similar to the observed population pyramid [33].

We calculated the log-likelihood of the data under each model by assuming that the reported number of weekly RVGE cases in each age group was Poisson distributed with a mean equal to the model-predicted number of cases (see Text S1 for details). The best-fitting parameter set was that which maximized the log-likelihood (log(*L*)) of the age-stratified time series for the given set of estimated and fixed parameters. To compare across models, we calculated the Akaike information criterion (AIC) as 2k – 2log(L), where k is the number of estimated parameters (Table 3). Lower AIC values indicate a better model fit.

### Impact of vaccination

We incorporated vaccination into each of the best-fitting models and explored both the short- and long-term effects of vaccination under a variety of assumptions about vaccine coverage and efficacy. Our analysis focused on model projections for the incidence of severe RVGE (Vesikari score ≥11); results for any RVGE are presented in Text S1 and Figure S3.

Most of the models assume the effect of vaccination is comparable to natural immunity from rotavirus infection, as observed in natural history studies, such that successive doses of the vaccine confer immunity comparable to that conferred by one or more natural infections. Thus, vaccination was assumed to confer some protection against infection and stronger protection against infectiousness and disease given infection. This approach yields predicted vaccine efficacies against RVGE similar to those measured during clinical trials conducted in developed countries (see below) [34], [35], [36], [37]. For Model A, however, a separate input parameter was required for the vaccine efficacy, because risk of infection was assumed to decline with age rather than with the number of previous infections. We assumed the vaccine efficacy in Model A was equal to that predicted under the other models (see Text S1).

It is unclear whether successive doses of a vaccine confer additional protection, as is observed with natural infections. To address this uncertainty, we explored two possible scenarios: (1) vaccination confers protection comparable to that conferred by primary infection following the first dose administered at 2 months of age (with further doses providing no additional benefit), and (2) vaccination confers protection comparable to that following primary infection when the first dose is given at 2 months of age *and* additional protection comparable to that conferred by secondary infection when the second dose is administered at 4 months of age. We assumed that 96% of individuals receiving each dose of the vaccine seroconvert and therefore benefit from that dose of the vaccine [38] (i.e. protection is “leaky-or-nothing" [30], with 4% of individuals receiving no protection whatsoever). Scenario 1 equates to a vaccine efficacy of 82% against severe RVGE and 64% against any RVGE, whereas scenario 2 equates to vaccine efficacies of 99% and 74% against severe and any RVGE, respectively (see Text S1 for calculations). These values represent the breadth of vaccine efficacy estimates derived from clinical trials conducted in Europe and Latin America [34], [35], [36], [37].

In preliminary analyses, we also explored the effect of vaccination assuming that protection is comparable to that conferred by primary infection and only occurs after the second vaccine dose at 4 months of age (weakest effect), or allowing for additional vaccine-induced protection following each dose including a third vaccine dose administered at 6 months of age (strongest effect). However, model projections for the short- and long-term impact of vaccination under these scenarios did not differ substantially from those under scenarios 1 and 2, respectively.

For all models, we evaluated the short-term effect of vaccination on the dynamics of rotavirus over a five-year period following the introduction of the vaccine. For each scenario, we assumed that the vaccine was introduced into the population prior to the start of the rotavirus season in October (week 40) at a coverage level of either 70% or 90% of all eligible infants; coverage was maintained at a constant level following vaccine introduction. We examined model projections for the incidence of severe RVGE and any RVGE in children <5 years of age (an age range in which 95% of reported RVGE cases occur prior to vaccination) and in individuals ≥5 years of age to examine a possible shift in the burden of illness to older age groups following vaccine introduction.

We also examined the short-term percent reduction in the cumulative incidence of severe RVGE cases predicted by the models over one, two, and five years after the introduction of the vaccine (evaluated starting from week 1 of the year following vaccine introduction) and compared this to the direct effect of vaccination.

The long-term effect of vaccination was measured by the percent reduction in the mean incidence of severe or any RVGE over the full range of vaccine coverage levels (0–100%) during a 10 year period beginning 10 years after vaccine introduction, i.e. during years 10–19 post-introduction. Again, we compared this to the direct effect of the vaccine.

The direct effect of vaccination *y* years after vaccine introduction was calculated as the product of the assumed vaccine efficacy, the weekly proportion of vaccinated individuals in each age group, and the weekly number of RVGE cases reported pre-vaccination in that age group, summed over all age groups and weeks from week 1 of the year following vaccine introduction to week 52 of year *y* (see Text S1 for details). Thus, our calculation of the direct effect assumes that vaccinated individuals are the only ones to benefit from vaccination and are protected for life. The degree of indirect protection was determined by subtracting the direct effect from the overall reduction projected by the models.

## Results

**Figure 1:**
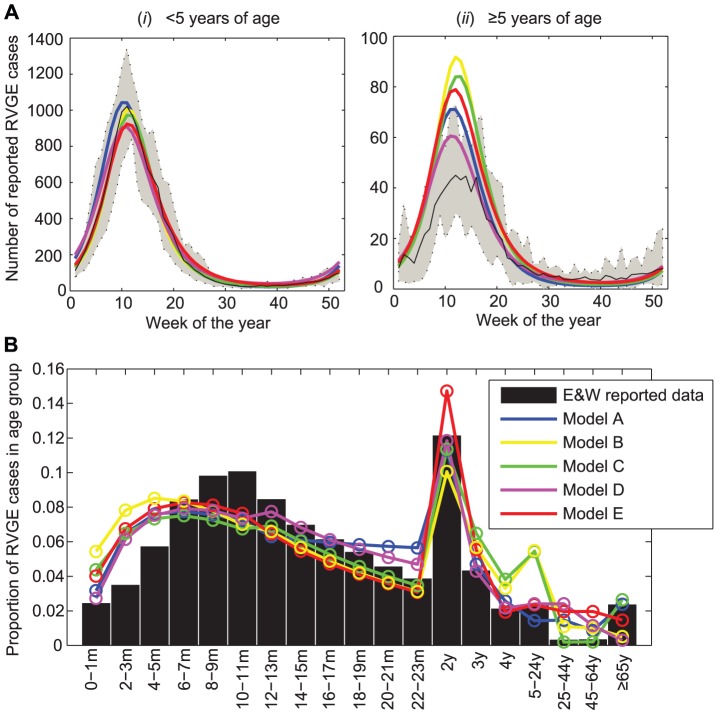
Fit of models to RVGE case reports from England and Wales. (A) Mean annual size and timing of rotavirus epidemics in individuals (i) <5 years of age and (ii) ≥5 years of age. The solid black line represents the mean number of RVGE cases per week. Dashed lines show the minimum and maximum number of cases each week. Colored lines represent the fitted models: Model A (blue), Model B (yellow), Model C (green), Model D (purple), Model E (red). (B) Age distribution of reported rotavirus cases (bars) and the fitted models (colored lines).

All the fitted models were qualitatively similar to the E&W rotavirus data (Figure 1, Figure S2), reproducing the pattern of strong winter seasonal epidemics occurring around week 9–13 each year (Figure 1A). The models also reproduced the age distribution of cases, although they tended to over-estimate the proportion of cases occurring in the youngest age groups (<6 months of age) and in either the 5–24 year (Models B and C) or 25–64 year (Models A, D, and E) age groups (Figure 1B).

### Short-term impact of vaccination

**Figure 2:**
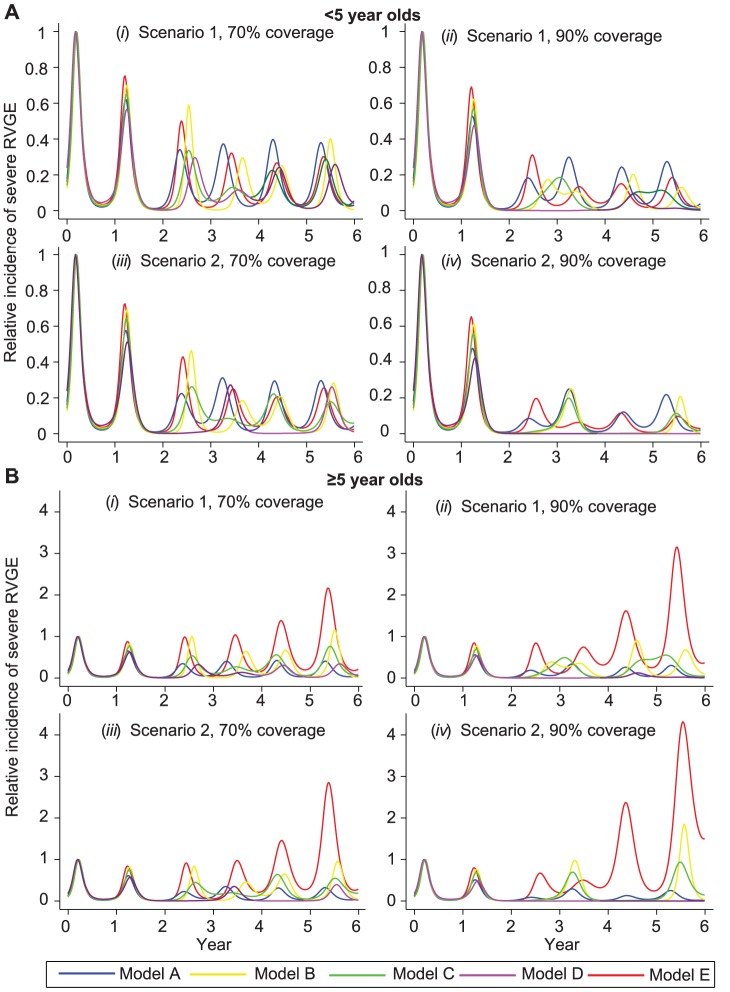
Short-term dynamics of rotavirus epidemics in the first 5 years after vaccine introduction. (A) Weekly incidence of severe RVGE predicted for individuals <5 years of age, scaled by peak pre-vaccination incidence, for the following scenarios: (i) 70% coverage with a vaccine that confers immunity comparable to primary infection following first dose at 2 months of age (82% efficacy) (scenario 1); (ii) 90% coverage under scenario 1; (iii) 70% coverage with a vaccine that confers immunity comparable to one natural infection following each dose at 2 and 4 months of age (99% efficacy) (scenario 2); and (iv) 90% coverage under scenario 2. (B) Incidence of severe RVGE predicted in individuals ≥5 years of age under coverage scenarios (i–iv) for Model A (blue), Model B (yellow), and Model C (green), Model D (purple), Model E (red).

The five models predicted a range of possible short-term dynamics following vaccine introduction (Figure 2). We examined the relative incidence of severe RVGE before and after vaccination and normalized the results by dividing by the peak pre-vaccination incidence. Under scenario 1 with vaccine coverage of 70%, the relative incidence of severe RVGE in children <5 years old was reduced by 25% (Model E) to 37% (Model D) in the first year following vaccine introduction, but the timing of the rotavirus epidemic was similar to the pre-vaccination timing (Figure 2A). The biggest discrepancies among the various model projections emerged during the second year following vaccine introduction. The peak in rotavirus incidence varied from week 19 (Model A) to week 36 (Model D). The peak incidence was reduced by 41–50% in Models B and E and by 66–70% in Models A, C, and D (Figure 2A,i), although differences in overall annual incidence were less dramatic. During the third year, peak incidence was reduced by 87–89% in Models C and D, but was only reduced by 63–71% in Models A, B, and E. Under scenario 1, at 90% coverage, the discrepancy among projections of different models was greater during the second and third year following vaccine introduction. Model D predicted a two-year period of very low incidence during which elimination of rotavirus from the population would likely occur; Model C predicted a pattern of biennial epidemics with incidence increasing through the summer of year 2 (and future even years) and peaking early in year 3 (odd years). In contrast, the other models predicted annual epidemics (Figure 2A,ii). Similar discrepancies also occurred under scenario 2. Model D predicted a pattern of biennial epidemics at 70% coverage and elimination of the rotavirus from the population at 90% coverage, whereas Models A–C and E predicted annual epidemics at 70% coverage and potentially biennial epidemics at 90% coverage (Figure 2A,iii–iv).

The incidence of severe RVGE in individuals older than 5 years exhibited similar timing to the incidence in children <5 years of age, but some of the models suggested that the relative incidence of severe RVGE in older individuals could increase following vaccine introduction (Figure 2B). The relative incidence of severe RVGE was greatest under Model E, increasing greater than four-fold at 90% coverage under scenario 2 (Figure 2B,iv). Models B and C also predicted an increase in the incidence of severe RVGE in this age group, but the relative increase was less than two-fold under these models.

**Figure 3:**
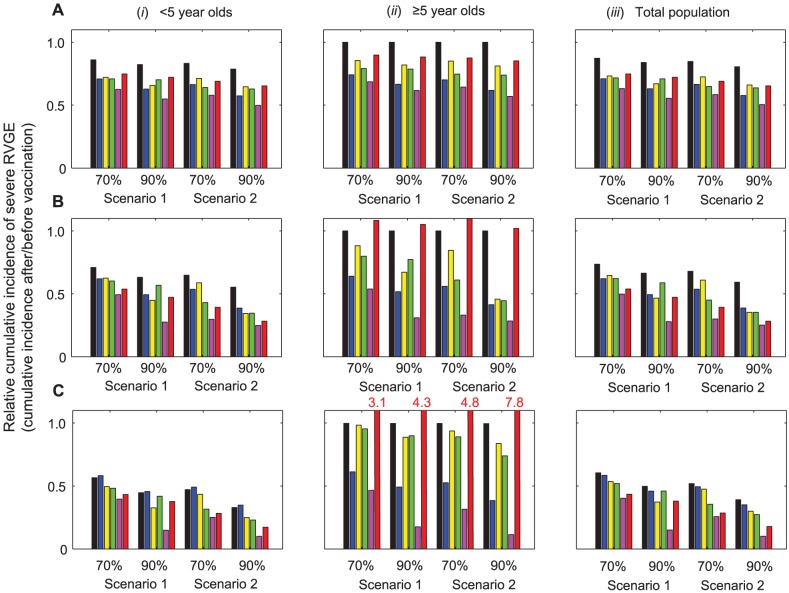
Short-term impact of vaccination on the cumulative incidence of severe RVGE predicted by the models. The relative cumulative incidence of severe RVGE after versus before vaccine introduction in individuals (i) <5 years of age, (ii) ≥5 years of age, and (iii) all age groups over the first (*A*) 1 year, (*B*) 2 years, and (*C*) 5 years after vaccine introduction are presented for scenarios 1 and 2 at 70% and 90% coverage. Black bars represent the direct effects of vaccination (see Text S1), while the colored bars represent the model projections: Model A (blue), Model B (yellow), Model C (green), Model D (purple), Model E (red). The *y*-axis is truncated at 1.1; where the bars exceed this threshold, i.e. for Model E in panel (*C*,*ii*), the red numbers indicate the relative cumulative incidence.

The reduction in the cumulative incidence of severe RVGE during the first year after vaccine introduction was similar across the five models, particularly for the <5 year old population where most of the cases occur (Figure 3A). The models predicted a 25–37% reduction in the number of severe RVGE cases during the first year after vaccine introduction at 70% coverage when no additional benefit from successive doses of the vaccine was assumed (scenario 1); only a 13% reduction in incidence would be expected from the direct effect of the vaccine alone. The discrepancy among model projections was slightly greater at 90% coverage, particularly when the second dose of the vaccine is assumed to provide additional immunity (scenario 2). At 90% coverage, a 34–50% reduction in severe RVGE was predicted by the models, with a 19% reduction expected from the direct effect of the vaccine. Thus, all of the models predicted considerable short-term indirect protection evident from a 1.8- to 2.9-fold greater than expected reduction in the incidence of severe RVGE during the first year after vaccine introduction, compared to when indirect effects are ignored.

However, differences in the level of indirect protection predicted by the different models were accentuated when examining the reduction in cumulative incidence of severe RVGE over 2 to 5 years following vaccine introduction (Figure 3B,C). Model D consistently predicted the greatest reductions in the cumulative incidence of severe RVGE, which were substantially greater than the reduction in incidence due exclusively to the direct effect of the vaccine for both the <5 year and ≥5 year old populations. Model E predicted similar reductions in the incidence of severe RVGE occurring in children <5 years of age as compared to Model D (particularly at 70% coverage), but it predicted a substantial increase in the relative incidence of severe RVGE occurring in individuals ≥5 years old, which was not predicted by the other models. Models A–C predicted more modest reductions in the cumulative incidence of severe RVGE 2 to 5 years after vaccine introduction. There was little or no indirect protection against severe RVGE predicted by Model A when considering the cumulative incidence of severe RVGE in all age groups over the 5 year period after vaccine introduction. Model B also predicted minimal levels of indirect protection over the 5 years, particularly at 70% coverage.

### Long-term impact of vaccination

**Figure 4:**
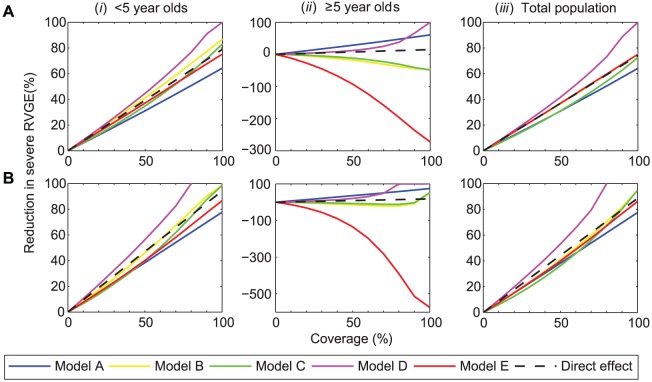
Long-term impact of vaccination on the incidence of severe RVGE predicted by the models. The reduction in the incidence of severe RVGE during a 10-year period beginning 10 years after vaccine introduction, as compared to the mean pre-vaccination incidence, is plotted for coverage levels from 0 to 100%. The panels represent the reduction in incidence of severe RVGE under (A) scenario 1: vaccination is assumed to confer immunity comparable to primary infection following the first dose at 2 months of age (82% efficacy), and (*B*) scenario 2: vaccination is assumed to confer immunity comparable to one natural infection following each dose at 2 and 4 months of age (99% efficacy), for (i) <5 years of age, (ii) ≥5 years of age, and (iii) all age groups. Black dashed lines represent the direct effect of vaccination (see Text S1), while solid colored lines represent the model projections: Model A (blue), Model B (yellow), Model C (green), Model D (purple), Model E (red).

With a vaccine efficacy of 82% against severe RVGE assumed under scenario 1, the predicted effects of vaccination range from a long-term reduction in the incidence of severe RVGE that is slightly less than would be expected from the direct effect of the vaccine alone to indirect protection that could eliminate rotavirus from the population at 100% coverage. Four of the five models, however, predicted that the vaccine would not provide long-term indirect protection in the population as a whole (Figure 4*A*,*iii*). Models B, C, and E show a reduction in the incidence of severe RVGE among children <5 years of age that exceeds that expected from the direct effect of the vaccine alone (Figure 4A,i), suggesting there may be some indirect protection for this age group, particularly at high coverage levels. However, this indirect protection was partially offset by an increase in the incidence of severe RVGE among individuals ≥5 years of age (Figure 4A,ii). Thus, Models B, C, and E suggest that vaccination will only delay the time to infection and disease in individuals not protected by the vaccine rather than preventing severe RVGE among these individuals. However, the overall reduction in severe RVGE predicted across all age groups due to the direct effect of vaccination is still substantial. Models A and D predicted a reduction in the incidence of severe RVGE among individuals ≥5 years old that indicates considerable indirect protection in this age group (Figure 4A,ii). However, for Model A, there was a less substantial reduction in incidence than expected based on the direct effects among children <5 years of age (Figure 4A,i).

The long-term impact of vaccination predicted by the models under scenario 2 was similar to that under scenario 1. However, because the efficacy of the vaccine against severe RVGE is assumed to be 99% under scenario 2 compared to 82% under scenario 1, the reduction in the incidence of severe RVGE due to the direct effect of vaccination is expected to be greater (Figure 4*B*). Again, most of the models predicted there would be little or no *additional* reduction in the incidence of severe RVGE (i.e. in excess of the direct effect) in the population as a whole, although Models B and C predicted there may be some indirect protection at coverage levels exceeding 90% (Figure 4B,iii). Elimination of rotavirus from the population was predicted to be possible under Model D when at least 80% of eligible infants were vaccinated with at least two doses of the vaccine (Figure 4B,iii). Model A, however, predicted only a 78% reduction in the incidence of severe RVGE at 100% coverage; this was due to a less substantial reduction in incidence than expected among <5 year olds (Figure 4B,i). Model E predicted a large increase in the relative incidence of severe RVGE among individuals ≥5 years old (Figure 4B,ii). Models B and C predicted a smaller increase in the relative incidence of severe RVGE among older individuals at coverage levels <90%, but a decline in incidence among ≥5 year olds at 100% coverage (Figure 4B,ii). However, all models predicted substantial declines in severe RVGE and all RVGE (Figure S3) for the population as a whole.

## Discussion

Our results reveal several interesting discrepant findings among the model projections for the short- and long-term impact of vaccination that shed light on some of the important questions about rotavirus epidemiology. Direct comparison of differences in model structure and estimated parameters allows us to understand the reasons for the variation in model projections. Fundamentally, these variations in model projections reflect gaps in our understanding of the mechanisms of rotavirus infection, natural immunity, epidemiology, and the biological nature of vaccine protection. Much of the uncertainty regarding the expected indirect effects of vaccination in the literature stems from different model assumptions for why severe RVGE is rare among older children and adults, and to what extent natural and vaccine-induced immunity wanes over time.

The five models we analyzed reproduce the seasonality and age distribution of rotavirus incidence in E&W, providing an important source of model validation. We compared the statistical fit of these models to week- and age-stratified laboratory reports of confirmed rotavirus cases from E&W, and found that all five models had AIC values between 66,697 and 84,997. Given the large number of data points we attempted to fit (547 weeks×19 age groups = 10,393 data points), the relatively large AIC values and the variation among models with different numbers of estimated parameters is not surprising. Model E provided the best fit to the pre-vaccination E&W RVGE reports, but the relative ranking of models based on AIC should be interpreted cautiously when extrapolating to the ability of the models to predict the impact of vaccination. Furthermore, we only explored a single set of fixed parameters and fit to a single data set; there is additional uncertainty regarding the parameter values that was not accounted for in our analysis, and which would presumably affect the fit differently for different models.

The models make different assumptions to explain why most RVGE cases occur among children <5 years of age (Table 1). Both Models A and D allow for the risk of infection and/or reporting to vary by age, and both models find that older individuals are less likely to be infected with rotavirus than younger individuals given equal levels of exposure (except among ≥65 year olds in Model A) (Table 3). These models are characterized by lower estimated values of the basic reproductive number *R*_0_ (quantifying the transmission potential of rotavirus), suggesting that the high incidence of rotavirus infection in the population is due to the repeated infection of individuals throughout their lifetime rather than being due to the large number of individuals who can be infected by a single individual with a primary infection. However, Models A and D differ in that Model A assumes that individuals ≥5 years of age are less prone to severe RVGE than those <5 years of age and therefore tend to be less infectious, whereas Model D assumes that severe infections occur in older individuals but tend to be under-reported at a higher rate. Furthermore, Model A assumes that immunity following natural infection and vaccination is not long-lasting and that the decline in the proportion of cases in older age groups is entirely due to age-specific differences in the risk of infection and disease. Model D assumes that immunity gained through repeated natural infections partially accounts for the greater concentration of RVGE in younger individuals. While Model A does not track asymptomatic infections and assumes they play no role in transmission, Model D does take into account possible transmission from such infections. Together, these differences help explain why the *R*_0_ for Model D is slightly greater than the *R*_0_ for Model A.

Models B, C, and E are similar to Model D in that they also assume the progressive build-up of natural immunity to both infection and symptoms of infection explains the greater concentration of RVGE in children <5 years of age. Immunity is assumed to wane on a time scale that does not affect the <5 year old population (Table 3). Unlike Model D, however, these models do not allow the rate of reporting of RVGE to differ between the <5 years old and ≥5 years old age groups. Thus, the estimated values of *R*_0_ for these models tend to be higher, suggesting a higher incidence rate of infection and younger age of first infection with rotavirus. As a result, there should be a greater correlation between the age distribution of reported RVGE cases and the RVGE cases responsible for the majority of rotavirus transmission predicted by Models B, C, and E. In contrast, Model D implicitly assumes that unreported cases of RVGE in older children and adults play an important role in transmission; paradoxically, this model projects stronger indirect protection because there are fewer overall infections among older individuals in Model D than in Models B, C, and E. In essence, Models A and D place more emphasis on adults in the transmission process compared with Models B, C, and E.

Part of the difficulty in deciding which model best represents the underlying epidemiology of rotavirus infection is lack of knowledge about the reporting pyramid for rotavirus. In other words, what fraction of rotavirus infections are symptomatic, what fraction of symptomatic infections present to the healthcare system, and what fraction of those presenting get properly diagnosed as rotavirus? A few studies have attempted to elucidate the reporting pyramid for rotavirus infections in E&W, but they were underpowered to understand the reporting fraction in adults, how reporting varies over time, and how it correlates with the severity of symptoms [27], [28], [29].

The similarities and differences in model structure are reflected in the projections that each model yields for the indirect protection conferred by vaccination. During the first 5 years after vaccine introduction, the reduction in the cumulative incidence of severe RVGE predicted by each model was similar for children <5 years of age and for the overall population. However, it is more difficult to predict the pattern of epidemics following the introduction of vaccination, as suggested by the different epidemic trajectories predicted by each model. Furthermore, none of the models account for additional complexities such as the interaction among different genotypes of rotavirus, or environmental or local effects. All of the models suggest that the post-vaccination timing of rotavirus activity can vary considerably, with possible peaks occurring in the summer and/or fall as opposed to the typical pre-vaccination peaks occurring in winter/spring. This is consistent with the relatively low amplitude of seasonal forcing (4.3–6.4%) estimated for each model, which suggests that environmental factors such as temperature or humidity only have a small effect on the transmission rate, and that the large RVGE epidemics evident in E&W primarily result from the dynamic interaction between susceptible and infectious individuals [10], [39]. By altering the rate at which fully susceptible infants enter the population, vaccination can lead to significant changes in the timing of rotavirus activity; thus, models of the transmission dynamics of rotavirus can aid in our understanding of the observed incidence patterns [3], [5], [40], [41], [42].

Model projections suggest the short-term reduction in the incidence of reported RVGE during the first five years after vaccine introduction will exceed estimates that account only for the direct effects of vaccination. This is supported by recent observations of the early impact of vaccination in countries that have introduced routine immunization, where 22–68% decreases in the incidence of RVGE have been reported in age groups not eligible to receive the vaccine [1], [2], [3], [4], [6], including older children and adults [43], [44]. At three sentinel sites in the US, the overall incidence of hospitalization for RVGE among children 6–11 months of age was reduced by 87% in 2008, when coverage among this age group was 77%; but in 2009 the RVGE hospitalization rate among <3 year olds was similar to that expected based on coverage and vaccine effectiveness estimates [45]. Models B, C, and E predict that the indirect benefits of vaccination during the short term may not extend to the long term, as the burden of RVGE may shift to older age groups. Beginning in the second year after vaccine introduction and continuing to the long term, model projections differ considerably for the ≥5 year old population.

All of the models predict that vaccination will lead to a delay in the average age of first infection with rotavirus and a decrease in the number of infections experienced by a single individual during his/her lifetime (results not shown). For Models B, C, and E which assume that the severity of RVGE is associated only with the number of previous infections, the delay in the time to infection may shift some of the burden of severe RVGE to older individuals, because first and second infections may be more likely to occur after 5 years of age. If infections tend to be less severe and/or less likely to be reported in the ≥5 year old age group than in the <5 year old group, as estimated by Models A and D, the delay in the time to infection is not predicted to increase the burden of RVGE in the ≥5 year old age group. Furthermore, the decrease in the overall number of infections experienced by individuals during their lifetime could translate into a decrease in the number of severe RVGE cases among older individuals, particularly if the lifetime number of infections predicted by the model is relatively small.

Whether or not vaccination provides long-term indirect protection against severe RVGE in children <5 years of age depends on whether vaccine-induced immunity wanes completely after 1 year, or if vaccinated infants remain at reduced risk of RVGE for a prolonged period of time. The discrepancy in results between Model A, which predicts the smallest reduction in severe RVGE, and Model D, which predicts that elimination of RVGE is possible under some scenarios, is due primarily to the different assumptions made regarding the duration of immunity. If one makes the alternative assumption in Model A that vaccine-induced immunity lasts at least 3 years, then in this case Model A also predicts that rotavirus could be eliminated at high coverage rates [8]. Models B and C assume that the effect of vaccination may wane partially after 1 year, but that vaccine-induced immunity does not wane completely, whereas Model E assumes that long-lasting immunity is only generated in some individuals. Determining if vaccine-induced immunity wanes over time, how waning occurs, and how it differs across populations, is an important avenue for future research.

An important limitation of this study is that we do not comprehensively explore the influences of parameter uncertainty. Key uncertainties in our fixed parameter assumptions for rotavirus include the duration of natural and vaccine-induced immunity, protection conferred by previous infection(s), and relative infectiousness of primary and subsequent symptomatic and asymptomatic) infections. Further work is needed to characterize and explore the impact of this parameter uncertainty [46], while epidemiologic studies are needed to obtain better estimates of unknown parameters.

### Conclusions

Overall, the comparison of these different models of rotavirus transmission and vaccination allowed us to examine the impact of structural uncertainty on the robustness of model projections, as well as identify key gaps in our understanding of rotavirus epidemiology. The models we compared suggest vaccination will lead to a 64–100% reduction in the incidence of severe RVGE and a 55–100% reduction in any RVGE at full coverage 10–20 years following vaccine introduction if vaccination confers protection comparable to a single natural infection (Figure 4, Figure S3). Similarly, vaccination will result in a 78–100% reduction in severe RVGE and a 70–100% reduction in any RVGE if a second dose of vaccine confers additional protection. How the reduction in the incidence of severe and any RVGE translates to fewer cases of reported RVGE will depend on whether reporting reflects only cases of severe RVGE or both mild and severe RVGE. Indirect protection against RVGE apparent in the first few years after vaccine introduction may or may not extend to the long term. Whether it is possible to eliminate rotavirus infection from the population depends critically on the transmissibility of primary infections (as indicated by the estimated *R*_0_ of the best-fit models), what fraction of cases goes unreported, and whether immunity wanes over time.

Our comparative analysis of the model projections for the indirect effects of vaccination identified three key questions that should be addressed to improve the accuracy of model predictions:

- What is the **role of adults** in rotavirus transmission? Infection with rotavirus later in life is typically asymptomatic and/or unreported, but infected individuals could be transmitting rotavirus to others. The models we explored differed in the emphasis placed on transmission from older children and adults, which could account for some of the differences in projections of the long-term impact of vaccination.
- What is the effect of **multiple vaccine doses** on protection? Vaccination appears to confer immunity similar to that of natural infection, but it is unclear whether multiple doses of the vaccine yield an added benefit. We explored a variety of different assumptions regarding the impact of multiple vaccine doses, and found that the indirect benefits of vaccination may be greater if each dose confers immunity comparable to an additional natural infection. Understanding whether additional doses of vaccine provide additional protection could lead to new dosing schedules for developing countries, where vaccine efficacy is lower.
- Does **vaccine-induced immunity wane** over time? If so, what is the nature of this waning of immunity, i.e. is it complete or incomplete? The models we explored make different assumptions about the possible waning of immunity, from complete waning of vaccine-induced immunity to no waning of immunity. Age-specific estimates of vaccine efficacy and case-control studies of vaccine effectiveness during the second year of life suggest that there may be some waning of vaccine-induced immunity [34], [35], [36], [37], particularly in developing country settings [47], [48], [49], [50], but interpretation of this data is complicated.

Experimental studies of rotavirus pathogenesis, carefully designed epidemiologic studies, and stronger statistical links between data and models will lead to better-informed model assumptions and help to discriminate among models. In addition, further validation and fitting of transmission dynamic models to post-vaccination data from different countries will help to refine model parameter estimates and improve projections of the long-term impact of vaccination.

## Supporting Information

Figure S1 **Compartmental diagrams detailing model structures.** Compartmental diagrams detailing the model structures. (*A*) Model A, (*B*) Model B, (*C*) Model C, (*D*) Model D, (*E*) Model E. The red lines indicate the effect of vaccination for two doses of vaccine under scenario 2.
(PDF)

Figure S2 **Time series of reported RVGE cases from England and Wales and fitted models, January 1999 to June 2009.** The reported number of RVGE cases per week among <5 year olds, ≥5 year olds, and the total population are plotted along with the fitted models from January 1999 to June 2009. Black lines represent the reported E&W data, while the colored lines represent the model projections: Model A (blue), Model B (yellow), Model C (green), Model D (purple), Model E (red).
(PDF)

Figure S3 **Long-term impact of vaccination on the incidence of any RVGE predicted by the models.** The reduction in the incidence of any RVGE during a 10-year period beginning 10 years after vaccine introduction, as compared to the mean pre-vaccination incidence, is plotted for coverage levels from 0 to 100%. The panels represent the reduction in incidence of any RVGE under (A) scenario 1: vaccination is assumed to confer immunity comparable to primary infection following the first dose at 2 months of age (64% efficacy), and (*B*) scenario 2: vaccination is assumed to confer immunity comparable to one natural infection following each dose at 2 and 4 months of age (74% efficacy), for (i) <5 years of age, (ii) ≥5 years of age, and (iii) all age groups. Black dashed lines represent the direct effect of vaccination, while solid colored lines represent the model projections: Model A (blue), Model B (yellow), Model C (green), Model D (purple), Model E (red).
(PDF)

Table S1 **Fixed parameter values for five models, for both the original publication and the current analysis.**
(PDF)

Text S1 **Supporting Text.** Details on the parameter estimates, model fitting procedure, calculation of the direct effect of vaccination and vaccine efficacy, and results for the long-term impact of vaccination in the incidence of any RVGE.
(PDF)
