# Supplementary figures and images for: Direct and Indirect Effects of Rotavirus Vaccination: Comparing Predictions from Transmission Dynamic Models
Source: PLoS One. 2012 Aug 13;7(8):e42320. doi: 10.1371/journal.pone.0042320 (PMC3418263; doi:10.1371/journal.pone.0042320)

**A**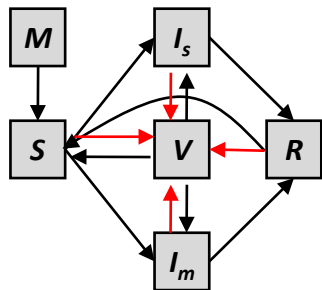**B**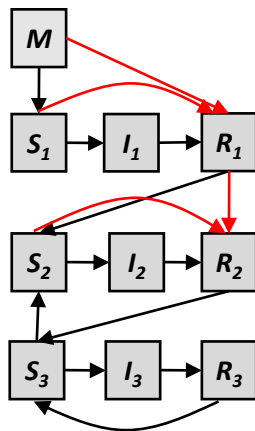**C**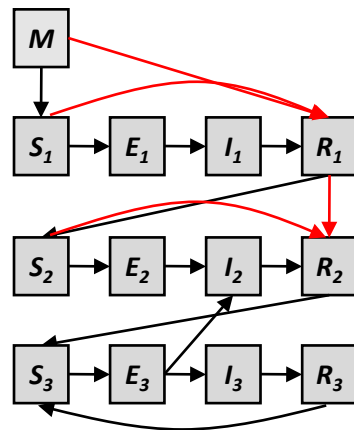**D**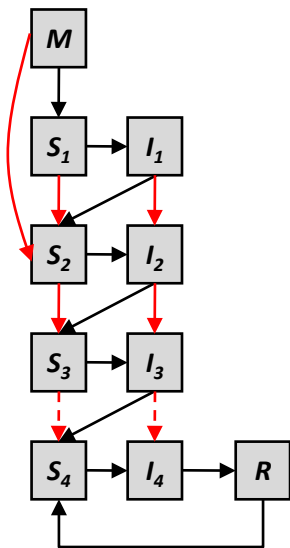**E**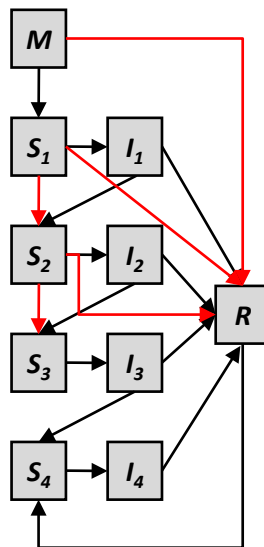

Supplement: Figure S1 — Compartmental diagrams detailing model structures. Compartmental diagrams detailing the model structures. (A) Model A, (B) Model B, (C) Model C, (D) Model D, (E) Model E. The red lines indicate the effect of vaccination for two doses of vaccine under scenario 2. (PDF) [file pone.0042320.s001.pdf]

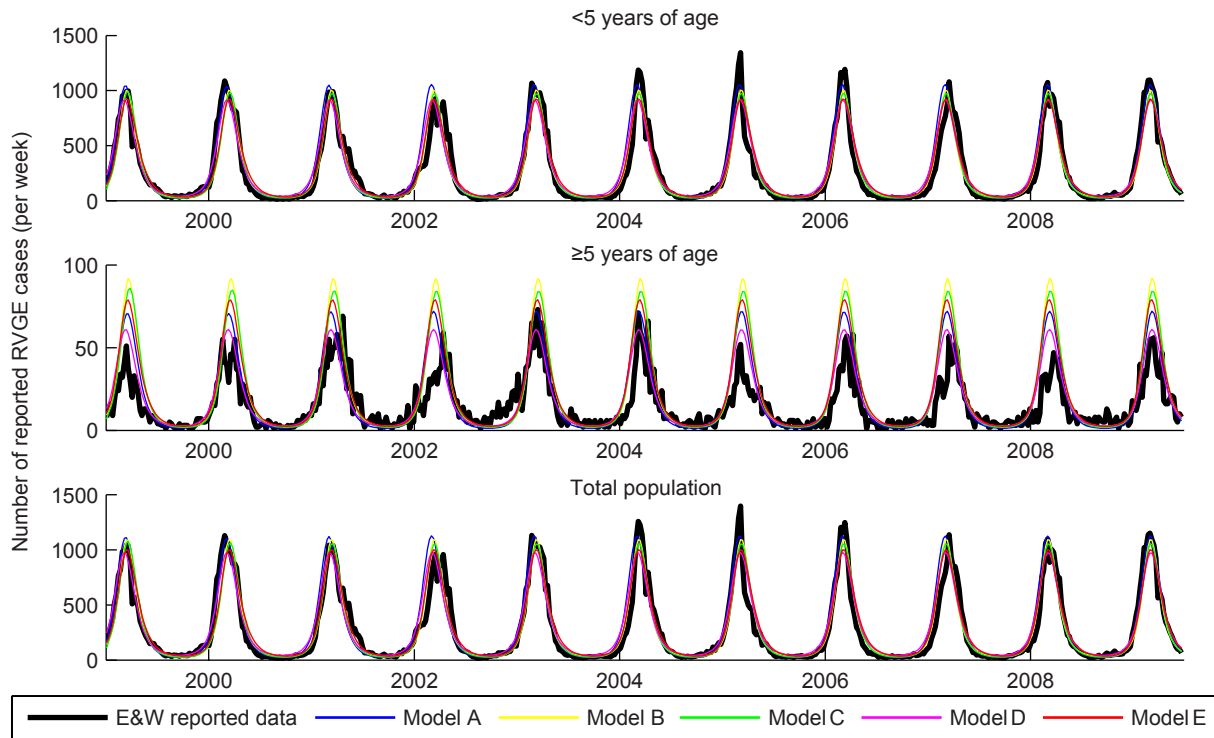

Supplement: Figure S2 — Time series of reported RVGE cases from England and Wales and fitted models, January 1999 to June 2009. The reported number of RVGE cases per week among <5 year olds, ≥5 year olds, and the total population are plotted along with the fitted models from January 1999 to June 2009. Black lines represent the reported E&W data, while the colored lines represent the model projections: Model A (blue), Model B (yellow), Model C (green), Model D (purple), Model E (red). (PDF) [file pone.0042320.s002.pdf]

**A**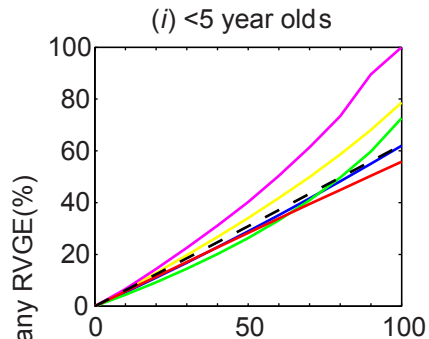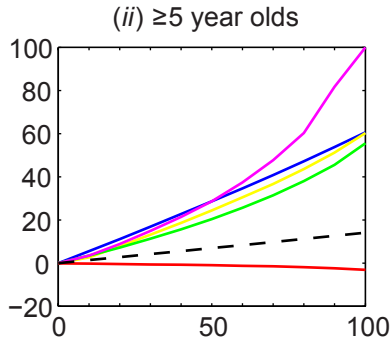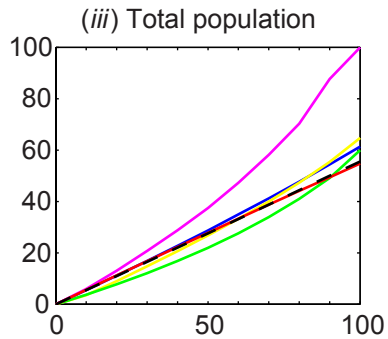**B**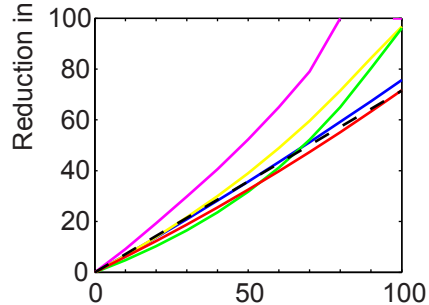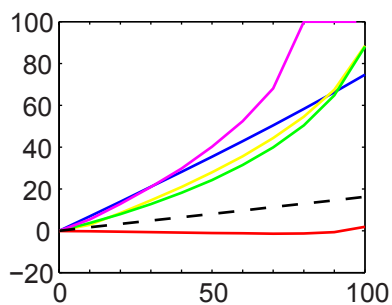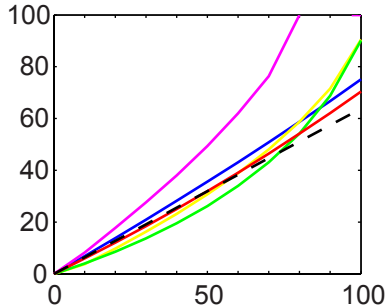

Coverage (%)

— Model A — Model B — Model C — Model D — Model E - - - Direct effect

Supplement: Figure S3 — Long-term impact of vaccination on the incidence of any RVGE predicted by the models. The reduction in the incidence of any RVGE during a 10-year period beginning 10 years after vaccine introduction, as compared to the mean pre-vaccination incidence, is plotted for coverage levels from 0 to 100%. The panels represent the reduction in incidence of any RVGE under (A) scenario 1: vaccination is assumed to confer immunity comparable to primary infection following the first dose at 2 months of age (64% efficacy), and (B) scenario 2: vaccination is assumed to confer immunity comparable to one natural infection following each dose at 2 and 4 months of age (74% efficacy), for (i) <5 years of age, (ii) ≥5 years of age, and (iii) all age groups. Black dashed lines represent the direct effect of vaccination, while solid colored lines represent the model projections: Model A (blue), Model B (yellow), Model C (green), Model D (purple), Model E (red). (PDF) [file pone.0042320.s003.pdf]
