# Supplementary material for: Direct and Indirect Effects of Rotavirus Vaccination: Comparing Predictions from Transmission Dynamic Models
Source: PLoS One. 2012 Aug 13;7(8):e42320. doi: 10.1371/journal.pone.0042320 (PMC3418263; doi:10.1371/journal.pone.0042320)
Supplement: Table S1 — Fixed parameter values for five models, for both the original publication and the current analysis. (PDF) [file pone.0042320.s004.pdf]

Table S1. Fixed parameter values for five models, for both the original publication and the current analysis.

Model [ref*]	Model A [18,19]		Model B [20]		Model C [21]		Model D [21]		Model E [23]	
**Parameter [ref]**	**Original**	**Current**	**Original**	**Current**	**Original**	**Current**	**Original**	**Current**	**Original**	**Current**
Duration of maternal immunity [5]	NA	13 weeks	3 months	13 weeks	90 days	13 weeks	40 days	13 weeks	90 days	13 weeks
Duration of incubation period [3]	NA	NA	NA	NA	1 day	1 day	NA	NA	NA	NA
Duration of infectiousness										
First infection [10,11]	5 days (severe)	7 days (severe)	7 days	7 days	8 days	7 days	10 days	7 days	8 days	7 days
Subsequent infections [8,9]	3 days (mild)	3.5 days (mild)	3.5 days	3.5 days	6 days for second, 4 days for subsequent	3.5 days	10 days	3.5 days	8 days	7 days
Relative risk of infection following: [7]										
First infection	NA	NA	0.62	0.62	0.62	0.62	0.62	0.62	0.62	0.62
Second infection			0.35	0.37	0.375	0.37	0.40	0.37	0.40	0.37
Third infection			0.35	0.37	0.375	0.37	0.34	0.37	0.37	0.37
Proportion of infections with any RVGE (severe RVGE) [7]										
First infection	0.76 mild, 0.24 severe for <5 yr olds	0.76 mild, 0.24 severe for <5 yr olds	NA (0.11)	0.47 (0.13)	0.47 (0.13)	0.47 (0.13)	0.39 (0.11)	0.47 (0.13)	0.47	0.47 (0.13)
Second infection			NA (0.029)	0.25 (0.03)	0.25 (0.03)	0.25 (0.03)	0.14 (0.03)	0.25 (0.03)	0.25	0.25 (0.03)
Third infection	1 mild, 0 severe for ≥5 yr olds	Estimated for ≥5 yr olds	NA (0)	0.20 (0)	0.20 (0)	0.20 (0)	0.17 (0)	0.32 (0)	0.32	0.32 (0)
Fourth infection			NA	NA	NA	NA	0.09 (0)	0.20 (0)	0.20	0.20 (0)
Relative infectiousness (compared to first infection) [2,4]										
Second infection	0.5 (mild vs severe)		0.5	0.5	0.5	0.5	Values between 1 and 0.25	0.5	Only individuals with symptomatic RVGE transmit (see above)	
Subsequent infections	Asymptomatic infections do not transmit		0.1	0.2	0.2	0.2		0.2		
Duration of complete immunity [1,6]	5 years	1 year	9 months to 1 year	1 year	2 months	1 year	NA	NA	NA	NA
Type of cases reported	Severe RVGE		Severe RVGE		Severe RVGE		Any RVGE		Any RVGE	

*ref=reference; numbers correspond to references in the Supporting Text.
