# Supplementary material for: Direct and Indirect Effects of Rotavirus Vaccination: Comparing Predictions from Transmission Dynamic Models
Source: PLoS One. 2012 Aug 13;7(8):e42320. doi: 10.1371/journal.pone.0042320 (PMC3418263; doi:10.1371/journal.pone.0042320)
Supplement: Text S1 — Supporting Text. Details on the parameter estimates, model fitting procedure, calculation of the direct effect of vaccination and vaccine efficacy, and results for the long-term impact of vaccination in the incidence of any RVGE. (PDF) [file pone.0042320.s005.pdf]

Supporting Text

Direct and Indirect Effects of Rotavirus Vaccination: Comparing Predictions from Transmission Dynamic Models

Virginia E. Pitzer[1,2*], Katherine E. Atkins[3†], Birgitte F. de Blasio[4,5†], Thierry Van Effelterre[6†], Christina J. Atchison[7], John P. Harris[8], Eunha Shim[9], Alison P. Galvani[3], W. John Edmunds[7], Cecile Viboud[2], Manish M. Patel[10], Bryan T. Grenfell[1,2], Umesh D. Parashar[10], Ben A. Lopman[10]

[1] *Department of Ecology and Evolutionary Biology, Princeton University, Princeton, NJ, USA*
[2] *Fogarty International Center, National Institutes of Health, Bethesda, MD, USA*
[3] *Yale School of Public Health, New Haven, CT, USA*
[4] *Department of Biostatistics, Institute of Basic Medical Sciences, University of Oslo, Oslo, Norway*
[5] *Department of Infectious Diseases Epidemiology, Norwegian Institute of Public Health, Oslo, Norway*
[6] *GlaxoSmithKline Biologicals, Wavre, Belgium*
[7] *Infectious Diseases Epidemiology Unit, Department of Epidemiology and Population Health, London School of Hygiene and Tropical Medicine, London, UK*
[8] *Centre for Infections, Department of Gastrointestinal, Emerging and Zoonotic Infections, Health Protection Agency, London, UK*
[9] *Deparment of Epidemiology, Graduate School of Public Health, University of Pittsburgh, Pittsburgh, PA, USA*
[10] *Epidemiology Branch, Division of Viral Diseases, National Center for Immunization and Respiratory Diseases, Centers for Disease Control and Prevention, Atlanta, GA, USA*

[*] E-mail: virginia.pitzer@yale.edu, Current address: Department of Epidemiology of Microbial Diseases, Yale School of Public Health, New Haven, CT, USA
[†] Equal contributors, listed alphabetically

Contents

1 Parameter estimates **3**
1.1 Fixed parameters . . . 3
1.2 Population mixing and transmission rates . . . 3

2 Detailed description of model fitting procedure **3**

3 Calculation of the direct effect of vaccination **5**

4 Calculation of the vaccine efficacy **6**

5 Long-term impact of vaccination on incidence of any RVGE **7**

1 Parameter estimates

1.1 Fixed parameters

Values of fixed parameters were derived from the published epidemiological literature [1–11]. Small discrepancies in parameter choices existed among the models, typically related in variation in interpretation of the literature or model time scales (i.e. choice of a daily, weekly, etc time step). Where such discrepancies existed, we chose a single (typically intermediate) parameter value to be used by all the models (Table S1). Where more substantial differences in fixed parameter choices existed related to the choice of model structure (Figure S1), we retained the original parameter values.

1.2 Population mixing and transmission rates

We assumed transmission-relevant population mixing reflected self-reported behavior on the number of physical contacts between individuals belonging to different age groups collected as part of the POLYMOD study [12]. We had data on the reported number of contacts among individuals in the following age groups: <1 year, 1-4 years, 5-14 years, 15-24 years, 25-44 years, 45-64 years, and ≥65 years old. We assumed the population mixing rate between age groups was proportional to the reported contact rate, while mixing within age groups was assumed to be homogeneous.

The mean transmission rate from an individual in age group j to an individual in age group i, denoted $\bar{\beta}_{i,j}$, was then calculated as the age-specific contact rate between individuals of age i and j ($C_{i,j}$) times the age-specific risk of infection for an individual in age group i (q_i):

$$\bar{\beta}_{i,j} = q_i C_{i,j}. \tag{1}$$

We incorporated seasonal forcing in the model by assuming the transmission rate varied sinusoidally with a period of one year (52 weeks):

$$\beta_{i,j}(t) = \bar{\beta}_{i,j}\left(1 + b\cos\left(\frac{2\pi t - 52\phi}{52}\right)\right), \tag{2}$$

where b is the amplitude of seasonal forcing, ϕ is the seasonal offset parameter (i.e. timing of peak transmission), and t is the time in weeks. The age-specific force of infection (incidence rate per fully susceptible individual) at time t, $\lambda_i(t)$, can then be calculated as

$$\lambda_i(t) = \sum_j \beta_{i,j}(t) Y_j(t), \tag{3}$$

where $Y_j(t)$ is the total number of infectious individuals in age group j at time t times their relative infectiousness, which will vary based on the infection number (first, second, etc) and/or severity, depending on the model structure (see Table 1).

2 Detailed description of model fitting procedure

The number of severe and mild RVGE cases occurring at time t was calculated as a fraction d_s and d_m, respectively, of all infections occurring among individuals in age group i (Model A) or infection number n (Models B-E), as follows:

Model A

$$\text{RVGE}_{severe}(t) = \sum_i d_{s,i} \lambda_i(t) S_i(t) \tag{4}$$

$$\text{RVGE}_{mild}(t) = \sum_i d_{m,i} \lambda_i(t) S_i(t) \tag{5}$$

Models B-E

$$\text{RVGE}_{severe}(t) = \sum_i \sum_n d_{s,n} \lambda_i(t) S_{n,i}(t) \tag{6}$$

$$\text{RVGE}_{mild}(t) = \sum_i \sum_n d_{m,n} \lambda_i(t) S_{n,i}(t) \tag{7}$$

where $S_{n,i}(t)$ represents the number of susceptible individuals in age group i (who have experienced $n-1$ previous infections, in Models B-E).

In fitting our models to the number of laboratory-confirmed rotavirus reports from England and Wales (E&W), we assumed that a fraction r of all severe RVGE (Models A-C) or any (severe and mild) RVGE cases (Models D-E) occurring at week t were reported in our dataset. For Model D, this reporting fraction r was assumed to vary for individuals <5 years versus ≥5 years of age, whereas for Model E, r was fixed at 0.029 [13]. Thus, the model-predicted number of reported RVGE cases in age group i at time t ($\hat{x}_i(t)$) for each of the models is given by:

Model A

$$\hat{x}_i(t) = r d_{s,i} \lambda_i(t) S_i(t) \tag{8}$$

Models B-C

$$\hat{x}_i(t) = r \sum_n d_{s,n} \lambda_i(t) S_{n,i}(t) \tag{9}$$

Model D

$$\hat{x}_i(t) = \begin{cases} r_{<5} \sum_n d_{m,n} \lambda_i(t) S_{n,i}(t) & \text{for } < 5 \text{ year olds} \\ r_{\geq 5} \sum_n d_{m,n} \lambda_i(t) S_{n,i}(t) & \text{for } \geq 5 \text{ year olds} \end{cases}$$

Model E

$$\hat{x}_i(t) = 0.029 \sum_n d_{m,n} \lambda_i(t) S_{n,i}(t) \tag{10}$$

We fit our models to the data by maximizing the log-likelihood of each model assuming the number of reported RVGE cases in age group i at week w ($x_{i,w}$, where w is the discrete-time equivalent of t) was Poisson-distributed with a mean equal to the model-predicted number of cases occurring at that time. The log-likelihood ($\log(L)$) of each model was given by the equation:

$$\log(L) = \sum_w \sum_i \left(-\hat{x}_i(t=w) + x_{i,w} \log \hat{x}_i(t=w) - \sum_{j=1}^{x_{i,w}} \log j \right), \tag{11}$$

We fit the models after an initial burn-in period long enough to reach the epidemiological steady state prior to vaccination. The length of the burn-in period was chosen to be divisible by 52 weeks so that the estimated seasonal offset parameter could be interpreted as the fraction of the year in which the transmission rate peaked. The time series of reported RVGE cases from E&W and fitted models from January 1999 to June 2009 are shown in Figure S2.

Since some of the models were originally fit by minimizing the sum of squares rather than the negative log-likelihood (Models D and E), we also fit these models using this method. The best-fit parameter sets were similar using both methods.

3 Calculation of the direct effect of vaccination

The direct effect of vaccination is the expected reduction in the incidence of severe (or any) RVGE resulting from the protection conferred by vaccination on vaccinated individuals, in the absence of any resulting reduction in transmission. The direct effect of vaccination on the *individual* can be estimated as $DE_{\text{indiv}} = 1 - AR_v/AR_u$, where AR_v is the attack rate among vaccinated individuals and AR_u is the attack rate among unvaccinated individuals in the same population, in which they are exposed to the same force of infection. This value is equivalent to the vaccine efficacy (VE) as it is conventionally measured during vaccine trials.

The direct effect of vaccination *in the population* can be defined as

$$DE_{\text{pop}} = 1 - x_{v,\lambda_0}(a,t)/x_u(a,t), \tag{12}$$

where $x_{v,\lambda_0}(a,t)$ is the incidence in a (partially) vaccinated population at time t given *no reduction in the force of infection*, and $x_u(a,t)$ is the incidence in an unvaccinated population. The incidence $x_{v,\lambda_0}(a,t)$ cannot be observed when vaccination is expected to reduce the force of infection, but it can be estimated by standardizing the individual effects to the population. The incidence among individuals of age a at time t in the counterfactual vaccinated population with coverage $v(a,t)$ is the weighted sum of the expected incidence among the unvaccinated proportion of the population and the expected incidence among the vaccinated proportion of the population reduced only by the direct effect of the vaccine:

$$\begin{aligned} x_{v,\lambda_0}(a,t) &= x_u(a,t)(1 - v(a,t))\,1 + x_u(a,t)\,v(a,t)(1 - DE_{\text{indiv}}) \\ &= x_u(a,t) - x_u(a,t)\,v(a,t)\,DE_{\text{indiv}} \end{aligned} \tag{13}$$

Thus, it is a function of the expected incidence in the absence of vaccination ($x_u(a,t)$), the vaccine coverage ($v(a,t)$), and the individual direct effect (DE_{indiv}).

The expected incidence in the absence of vaccination, $x_u(a,t)$, can be simulated using the models. However, this would yield a slightly different estimate of the population direct effect for each of the models. Given the relative stability of rotavirus dynamics in E&W over the past decade, for the sake of clarity we assumed that the average pre-vaccination RVGE seasonal incidence in age group i ($x_{i,w}$, to which the models were fit) is representative of the expected incidence in the absence of vaccination for $a \in i$ and week of the year $t = w_{pv}$.

The direct effect of vaccination for Model M across all age groups y years after vaccine introduction under scenario k can therefore be estimated as:

$$\begin{aligned} DE_{M,y,k} &= 1 - \frac{\int\int x_u(a,t)\,\mathrm{d}a\,\mathrm{d}t - \int\int x_u(a,t)v(a,t)\,DE_{\text{indiv}}\,\mathrm{d}a\,\mathrm{d}t}{\int\int x_u(a,t)\,\mathrm{d}a\,\mathrm{d}t} \\ &\approx \frac{\sum_{w=1}^{52y}\sum_i v_{i,w}x_{i,w_{pv}}\,VE_k}{\sum_{w=1}^{52y}\sum_i x_{i,w_{pv}}} \end{aligned} \tag{14}$$

where $w = 1$ represents the week of vaccine introduction, $v_{i,w}$ is the proportion of vaccinated individuals in age group i at week w, $x_{i,w_{pv}}$ is the reported number of RVGE cases in age group i during an average pre-vaccination week w_{pv} (where w and w_{pv} represent the same week of the year), and VE_k is the assumed vaccine efficacy for scenario k (see Section 4). The population direct effects estimated using the model-simulated incidence ($x_u(a,t) = \hat{x}_i(t)$) were similar to those estimated using the observed incidence ($x_{i,w}$) and to one another for each of the models (results not shown).

The indirect effect of vaccination *in the population* is defined as expected reduction in incidence among both vaccinated and unvaccinated individuals in a (partially) vaccinated population due to the reduced force of infection, while total effect of vaccination is the reduction in incidence due to both the direct effect of vaccination on vaccinated individuals and the reduced force of infection, i.e. the sum of the

direct and indirect effects. The total effect of vaccination in the population predicted by Model M y years after vaccine introduction under scenario k can thus be estimated directly from the model output:

$$\begin{aligned} TE_{M,y,k} &= \frac{\int\int x_v(a,t)\ \mathrm{d}a\ \mathrm{d}t}{\int\int x_u(a,t)\ \mathrm{d}a\ \mathrm{d}t} \\ &\approx \frac{\sum_{w=1}^{52y}\sum_i \hat{x}_i(t=w)}{\sum_{w=1}^{52y}\sum_i x_{i,w_{pv}}} \end{aligned} \tag{15}$$

The indirect effect of vaccination for Model M y years after vaccine introduction under scenario k is then simply $IE_{M,y,k} = TE_{M,y,k} - DE_{M,y,k}$.

4 Calculation of the vaccine efficacy

For Models B-E, vaccination was assumed to impart immunity comparable to natural infection(s). Thus, vaccine efficacy was not a model input, but instead was implicitly derived from assumptions about the build-up of natural immunity. The assumed vaccine efficacy can be calculated as a function of the proportion who seroconvert to each dose, the reduction in the risk of any infection, and the relative risk of (severe) symptomatic infection. For scenario 1, in which we assumed that vaccination conferred immunity comparable to a single natural infection following first dose at 2 months of age, the vaccine efficacy against severe and any RVGE was calculated as:

$$VE_{1,severe} = 1 - \left[0.04(1) + 0.96\left(0.62 * \frac{0.03}{0.13}\right)\right] = 82.3\% \tag{16}$$

$$VE_{1,mild} = 1 - \left[0.04(1) + 0.96\left(0.62 * \frac{0.25}{0.47}\right)\right] = 64.3\% \tag{17}$$

where 0.04 represents the proportion of vaccinees who do NOT seroconvert to vaccination and 0.96 represents the proportion of vaccines who do serocovert; these proportions are multiplied by the relative incidence of RVGE in individuals with zero or one previous natural infection, respectively (Table S1).

For scenario 2, in which we assumed that vaccination conferred immunity comparable to primary infection following first dose and immunity comparable to second infection following second dose, the vaccine efficacy against severe and any RVGE was calculated by summing the products of the proportion of individuals who seroconvert to zero, one, or two doses times the expected reduction in RVGE incidence for the corresponding number of previous infections (Table S1), as follows:

$$VE_{1,severe} = 1 - \left[0.04^2(1) + 2(0.04)(0.96)\left(0.62 * \frac{0.03}{0.13}\right) + 0.96^2\left(0.37 * \frac{0}{0.13}\right)\right] = 98.7\% \tag{18}$$

$$VE_{1,mild} = 1 - \left[0.04^2(1) + 2(0.04)(0.96)\left(0.62 * \frac{0.25}{0.47}\right) + 0.96^2\left(0.37 * \frac{0.32}{0.47}\right)\right] = 74.1\% \tag{19}$$

These calculated vaccine efficacies were very similar to the range of estimates derived from randomized trials conducted in Europe and Latin America [14–17].

We chose to calculate the expected direct effect of vaccination using the observed pre-vaccination incidence rather than our best-fit models to minimize the variation between the direct effects expected for each of the models. Nevertheless, there were small variations among the expected direct effects calculated for each of the models resulting from differences in assumptions regarding whether reported cases reflected severe or any RVGE, and whether the fraction of cases that developed severe RVGE (Model

A) or reported (Model D) varied by age. These differences were generally <7% of the mean direct effect calculated for all models; thus, we only present the mean value calculated for all the models.

Model A assumes that the 82.3% and 98.7% modeled vaccine efficacies against severe RVGE (under scenarios 1 and 2, respectively), which are used to calculate the direct effects, pertain only to the first year of follow-up. If one accounts for this by assuming those ≥1 year of age no longer benefit from the direct protection of the vaccine, then in fact the direct effect of vaccination should be considerably less than if we assume direct protection from the vaccine is lifelong, and vaccination does in fact offer indirect protection relative to this reduced direct effect according to Model A [18].

5 Long-term impact of vaccination on incidence of any RVGE

The long-term impact of vaccination on the incidence of any (mild and severe) RVGE 10-20 years after vaccine introduction were similar to the results for severe RVGE (presented in the main text), although in most cases the relative reductions were less substantial (Figure S3). This is to be expected since the efficacy of the vaccines against mild RVGE is assumed to be lower than the efficacy against severe RVGE, based on natural history data [7] and consistent with data from randomized trials [14–17]. Thus, the direct effects against any RVGE that we calculated were lower than those for severe RVGE (Figure S3). However, Models B and C predicted substantially stronger indirect protection against any RVGE compared to severe RVGE, particularly for the ≥5 year old population. Model A also predicted stronger indirect protection against any RVGE for the population as a whole, as did Model E under scenario 2 (Figure S3). Model D again predicted the strongest overall effect of vaccination, with elimination of RVGE possible at $\sim$ 100% coverage under Scenario 1 and $\sim$ 80% coverage under Scenario 2.

Model E predicted a much smaller increase in the relative incidence of any RVGE (compared to severe RVGE) among ≥5 year olds (Figure S3, Figure 4 of the main text). The increase in any RVGE among ≥5 year olds predicted by Model E was similar to that for the relative incidence of severe RVGE predicted by Models B and C (Figure S3, Figure 4 of the main text). Therefore the magnitude of the relative increase in reported cases of RVGE among ≥5 year olds should be similar according to Models B, C, and E, since Model E assumes reported cases reflect the incidence of any RVGE, while Models B and C assume reported cases reflect only severe cases of RVGE.

References

1. Chiba S, Yokoyama T, Nakata S, Morita Y, Urasawa T, et al. (1986) Protective effect of naturally acquired homotypic and heterotypic rotavirus antibodies. Lancet 2: 417-21.

2. Kang G, Iturriza-Gomara M, Wheeler JG, Crystal P, Monica B, et al. (2004) Quantitation of group a rotavirus by real-time reverse-transcription-polymerase chain reaction: correlation with clinical severity in children in south india. J Med Virol 73: 118-22.

3. Kapikian AZ, Wyatt RG, Levine MM, Yolken RH, VanKirk DH, et al. (1983) Oral administration of human rotavirus to volunteers: induction of illness and correlates of resistance. J Infect Dis 147: 95-106.

4. Koopman JS, Monto AS, Longini J I M (1989) The tecumseh study. xvi: Family and community sources of rotavirus infection. Am J Epidemiol 130: 760-8.

5. Linhares AC, Gabbay YB, Freitas RB, da Rosa ES, Mascarenhas JD, et al. (1989) Longitudinal study of rotavirus infections among children from belem, brazil. Epidemiol Infect 102: 129-45.

6. Makela M, Marttila J, Simell O, Ilonen J (2004) Rotavirus-specific t-cell responses in young prospectively followed-up children. Clin Exp Immunol 137: 173-8.

7. Velazquez FR, Matson DO, Calva JJ, Guerrero L, Morrow AL, et al. (1996) Rotavirus infections in infants as protection against subsequent infections. N Engl J Med 335: 1022-8.

8. Ward RL, Bernstein DI, Young EC, Sherwood JR, Knowlton DR, et al. (1986) Human rotavirus studies in volunteers: determination of infectious dose and serological response to infection. J Infect Dis 154: 871-80.

9. Ward RL, Bernstein DI, Shukla R, McNeal MM, Sherwood JR, et al. (1990) Protection of adults rechallenged with a human rotavirus. J Infect Dis 161: 440-5.

10. White LJ, Buttery J, Cooper B, Nokes DJ, Medley GF (2008) Rotavirus within day care centres in oxfordshire, uk: characterization of partial immunity. J R Soc Interface .

11. Wilde J, Yolken R, Willoughby R, Eiden J (1991) Improved detection of rotavirus shedding by polymerase chain reaction. Lancet 337: 323-6.

12. Mossong J, Hens N, Jit M, Beutels P, Auranen K, et al. (2008) Social contacts and mixing patterns relevant to the spread of infectious diseases. PLoS Med 5: e74.

13. Wheeler JG, Sethi D, Cowden JM, Wall PG, Rodrigues LC, et al. (1999) Study of infectious intestinal disease in england: rates in the community, presenting to general practice, and reported to national surveillance. the infectious intestinal disease study executive. BMJ 318: 1046-50.

14. Linhares AC, Velazquez FR, Perez-Schael I, Saez-Llorens X, Abate H, et al. (2008) Efficacy and safety of an oral live attenuated human rotavirus vaccine against rotavirus gastroenteritis during the first 2 years of life in latin american infants: a randomised, double-blind, placebo-controlled phase iii study. Lancet 371: 1181-9.

15. Ruiz-Palacios GM, Perez-Schael I, Velazquez FR, Abate H, Breuer T, et al. (2006) Safety and efficacy of an attenuated vaccine against severe rotavirus gastroenteritis. N Engl J Med 354: 11-22.

16. Vesikari T, Karvonen A, Prymula R, Schuster V, Tejedor JC, et al. (2007) Efficacy of human rotavirus vaccine against rotavirus gastroenteritis during the first 2 years of life in european infants: randomised, double-blind controlled study. Lancet 370: 1757-63.

17. Vesikari T, Matson DO, Dennehy P, Van Damme P, Santosham M, et al. (2006) Safety and efficacy of a pentavalent human-bovine (wc3) reassortant rotavirus vaccine. N Engl J Med 354: 23-33.

18. Atkins KE, Shim E, Pitzer VE, Galvani AP (2012) Impact of rotavirus vaccination on epidemiological dynamics in england and wales. Vaccine 30: 552-64.

19. Shim E, Galvani AP (2009) Impact of transmission dynamics on the cost-effectiveness of rotavirus vaccination. Vaccine 27: 4025-30.

20. Pitzer VE, Viboud C, Simonsen L, Steiner C, Panozzo CA, et al. (2009) Demographic variability, vaccination, and the spatiotemporal dynamics of rotavirus epidemics. Science 325: 290-4.

21. de Blasio BF, Kasymbekova K, Flem E (2010) Dynamic model of rotavirus transmission and the impact of rotavirus vaccination in kyrgyzstan. Vaccine 28: 7923-32.

22. van Effelterre T, Soriano-Gabarro M, Debrus S, Claire Newbern E, Gray J (2010) A mathematical model of the indirect effects of rotavirus vaccination. Epidemiol Infect 138: 884-97.

23. Atchison C, Lopman B, Edmunds WJ (2010) Modelling the seasonality of rotavirus disease and the impact of vaccination in england and wales. Vaccine 28: 3118-26.
